# Prevalence of Metabolic Syndrome Is Higher among Non-Obese PCOS Women with Hyperandrogenism and Menstrual Irregularity in Korea

**DOI:** 10.1371/journal.pone.0099252

**Published:** 2014-06-05

**Authors:** Min-Ju Kim, Nam-Kyoo Lim, Young-Min Choi, Jin-Ju Kim, Kyu-Ri Hwang, Soo-Jin Chae, Chan-Woo Park, Doo-Seok Choi, Byung-Moon Kang, Byung-Seok Lee, Tak Kim, Hyun-Young Park

**Affiliations:** 1 Division of Cardiovascular and Rare Diseases, Center for Biomedical Science, Korea National Institute of Health, Chungbuk, Korea; 2 Department of Obstetrics and Gynecology, Seoul National University College of Medicine, Seoul, Korea; 3 Institute of Reproductive Medicine and Population, Medical Research Center, Seoul National University College of Medicine, Seoul, Korea; 4 Healthcare System Gangnam Center, Seoul National University Hospital, Seoul, Korea; 5 Seoul National University Boramae Medical Center, Seoul, Korea; 6 Department of Obstetrics and Gynecology, Maria Fertility Hospital, Seoul, Korea; 7 Department of Obstetrics and Gynecology, Cheil General Hospital and Women's Healthcare Center, Seoul, Korea; 8 Department of Obstetrics and Gynecology, Seoul Samsung Hospital, Sungkyunkwan University School of Medicine, Seoul, Korea; 9 Department of Obstetrics and Gynecology, Asan Medical Center, University of Ulsan College of Medicine, Seoul, Korea; 10 Department of Obstetrics and Gynecology, Gangnam Severance Hospital, Yonsei University College of Medicine, Seoul, Korea; 11 Department of Obstetrics and Gynecology, Korea University College of Medicine, Seoul, Korea; CHA University, Korea, Republic Of

## Abstract

**Background:**

Hyperandrogenism (HA) has been linked with several components of metabolic syndrome (MetS). Few studies in Asian women have evaluated the important risk factors for and prevalence of MetS according to PCOS subtype. In this study, we investigated differences in metabolic parameters and the prevalence of MetS in two major phenotypic subgroups of PCOS in Korea. Furthermore, we investigated the relationship between HA-associated parameters and MetS.

**Materials and Methods:**

This cross-sectional observational study was conducted from May 2010 to December 2011 in Korea. A total of 837 females with PCOS, aged 15–40, were recruited from Departments of Obstetrics and Gynecology at 13 hospitals. Of those, 700 subjects with either polycystic ovaries (PCO)+HA+oligomenorrhea/amenorrhea (O) or PCO+O were eligible for this study. MetS was diagnosed according to the modified National Cholesterol Education Program (NCEP) Adult Treatment Panel (ATP) III guidelines and the International Diabetes Federation (IDF) criteria.

**Results:**

MetS was more prevalent in the PCO+HA+O group (19.7%) than in the PCO+O (11.9%) group. There were statistically significant trends for an increased risk of MetS in the PCO+HA+O group compared to the PCO+O group. After adjustment for age, the odds ratio of MetS was 2.192 in non-obese subjects with PCO+HA+O compared to those with PCO+O, whereas the risk of MetS was not different in obese patients. Multivariate logistic regression analysis showed that high free androgen index and low sex hormone-binding globulin were significantly associated with MetS in non-obese women with PCOS, with odds ratios of 4.234 (95% CI, 1.893–9.474) and 4.612 (95% CI, 1.978–10.750), respectively. However, no associations were detected between MetS and SHBG and FAI in obese PCOS subjects.

**Conclusions:**

Our results indicate that HA and its associated parameters (FAI and SHBG) are significantly associated with MetS in non-obese PCOS subjects, whereas this association was not observed in obese subjects.

## Introduction

Polycystic ovary syndrome (PCOS) is a common endocrine disorder, affecting 5–10% of reproductive-aged women [1]–[3]. It is associated with metabolic features such as insulin resistance (IR), metabolic syndrome (MetS), dyslipidemia and increased cardiovascular risk factors [4]. The 2003 Rotterdam consensus workshop of the European Society for Human Reproduction and Embryology and the American Society for Reproductive Medicine (ESHRE/ASRM) proposed a definition of PCOS with at least two of the following three criteria: oligomenorrhea/amenorrhea (O), clinical or biochemical hyperandrogenism (HA), and polycystic ovaries (PCO) on ultrasonography [5]. According to these Rotterdam criteria, PCOS is divided into four phenotypes: PCO+HA+O, HA+O, PCO+O and HA+PCO [6].

MetS is a cluster of cardiovascular risk factors that are associated with glucose intolerance, dyslipidemia, obesity, and hypertension [7]. According to the modified National Cholesterol Education Program (NCEP) Adult Treatment Panal (ATP) III guidelines [8], the diagnosis of metabolic syndrome is made when three or more of the following risk factors are present: a waist circumference >102 cm in men and >88 cm in women, fasting plasma glucose ≥100 mg/dL, systolic blood pressure ≥130 mmHg or diastolic blood pressure ≥85 mmHg, fasting triglycerides ≥150 mg/dL, and high-density lipoprotein cholesterol (HDL-C) <40 mg/dL in men and <50 mg/dL in women. For Asian populations, the cut-off value for waist circumference was ≥90 cm in men and ≥80 cm in women based on the International Diabetes Foundation (IDF) [8].

IR with subsequent hyperinsulinemia plays a major role in the development of MetS [9]. In addition, IR plays an important role in the pathogenesis of PCOS [10]. Several studies have shown that women with PCOS have a higher prevalence of MetS than age-matched women in the general population [11]–[13]. The prevalence of MetS in women with PCOS has been reported to be 43.0–46.0% in the United States [11], [13]. In Korea, the prevalence of MetS in PCOS was 14.5%, nearly 3.5-fold higher than that reported for age-matched women in the Korean urban population [14], [15]. Hyperandrogenism (HA), a significant pathophysiological feature in PCOS, has been linked with several components of MetS [16]. In particular, androgen excess in PCOS may contribute to increased visceral fat, decreased lipolysis in subcutaneous fat, reduced insulin sensitivity in adipose tissue and skeletal muscle, decreased HDL-C levels, and increased low-density lipoprotein cholesterol (LDL-C) levels [16]. Some studies have indicated a positive association between MetS and HA in women with PCOS [11], [17]. Therefore, early and regular screening of metabolic disturbance in PCOS women is particularly important.

Although several studies have reported the independent risk factors for and prevalence of MetS in PCOS, few studies in Asian women have evaluated the important risk factors for and prevalence of MetS according to PCOS subtype. In this study, we examined differences in metabolic parameters and the prevalence of MetS in two major phenotypic subgroups of PCOS in Korea. Furthermore, we investigated the relationship between HA-associated parameters and MetS.

## Materials And Methods

### Ethics Statement

The study was approved by the Institutional Review Boards of Seoul National University, Seoul Samsung Hospital, Asan Medical Center, Severance Hospital, Gangnam Severance Hospital, Korea University Anam Hospital, Seoul National University Boramae Medical Center, MizMedi Hospital, Maria Fertility Hospital, Cheil General Hospital and Women's Healthcare Center, Pusan National University Hospital, Mirae-Heemang Hospital and Kyungpook National University Hospital. Written informed consent was obtained from all the subjects and/or their parents before participating in this study. Minors signed the written consent form by themselves in addition to their parent.

### Study participants

This cross-sectional observational study was conducted from May, 2010 to December, 2011 in Korea. In total, 837 women with PCOS were recruited from the Departments of Obstetrics and Gynecology at 13 hospitals. We selected 749 women with PCOS, aged 15–40, after excluding 88 subjects whose MetS component values were unavailable. PCOS was diagnosed according to the 2003 Rotterdam criteria as the presence of at least two of the following three features [5]: oligo or anovulation (O), clinical and/or biochemical HA and PCOs on ultrasound. Using these criteria, subjects with PCOS were divided into four subgroups: HA+PCO, HA+O, PCO+O and PCO+HA+O. Of the four different phenotypic groups, the PCO+HA+O (n = 432, 57.7%) and PCO+O (n = 268, 35.8%) groups were included in this analysis because the HA+PCO (n = 20, 2.7%) and HA+O (n = 29, 3.9%) groups were too small for statistical analysis. Thus, 700 subjects were eligible for this study. Oligo or anovulation was defined as a cycle length in excess of 35 days or less than 8 cycles per year or absence of menstruation for more than 3 months. Biochemical HA was defined as total testosterone (T) >0.68 ng/mL, free T>1.72 pg/mL, or a free androgen index (FAI) value >5.36, and a modified Ferriman–Gallwey score (mF-G score) of more than 6 was defined as clinical HA [18]. Ovaries were considered polycystic on ultrasound if there were 12 or more follicles measuring 2–9 mm in diameter in each ovary and/or enlarged ovarian volume (>10 mm^3^) [5]. Subjects with abnormal thyroid function, abnoromal prolactin levels, congenital adrenal hyperplasia, Cushing's syndrome, androgen-secreting tumors, diagnosed cardiovascular disease as well as those taking oral contraceptives, lipid-lowering agents, or insulin sensitizers were excluded from this study. This study was registered with the Clinical Research information Service (CRiS, registration number KCT0000739). Data were entered by a web-based case report form, using the clinical research and trial management system (iCReaT), which was developed by the Korea National Institute of Health.

### Clinical and biochemical measurements

Body weight and height were measured to the nearest 0.1 kg or 0.1 cm and body mass index (BMI) was calculated as body weight (kg) divided by height (meters) squared. Waist circumference (WC) was measured at the midpoint between the lower ribs and the top of the iliac crest in the standing position. Blood pressure (BP) was measured using a mercury sphygmomanometer after a 5-min rest period, and three BP readings were taken from right or left arms at 30-s intervals. The average systolic and diastolic BP values of two measurements taken in a sitting position were recorded.

Blood samples were collected after at least an 8-h fast. Fasting plasma glucose (FPG), total cholesterol (TC), triglyceride (TG), and HDL-C levels were measured enzymatically (Wako Pure Chemical Industries, Ltd. Osaka, Japan). The detection limit for FPG, TC, TG and HDL-C were 0–750 mg/dL, 0–675 mg/dL, 0–550 mg/dL and 17–90 mg/dL. Intra- and inter-assay coefficients of variation were 0-8-0.9% and 1.4–1.5% for FPG, 0.6–1.4% and 1.3–2.1% for TC, and 0.5–1.6%, 0.9–2.4% for TG and 0.6–0.7% and 2.2–2.5% for HDL-C. Fasting insulin levels were measured using a radioimmunoassay (RIA) (BioSource Europe S.A., Belgium) and Hemoglobin A_1C_ (HbA_1C_) was evaluated by immunoturbidimetric assay with a COBAS Integra 800 system (Roche Diagnostics, Basel, Switzerland). The homeostatic model for insulin resistance (HOMA-IR) was calculated by fasting insulin (µIU/mL) × fasting glucose (mg/dL)/(22.5×18).

Total T, free T and sex hormone-binding globulin (SHBG) concentrations were measured using a RIA (Siemens, Los Angeles, CA, USA) in the early follicular phase of the menstrual cycle or upon first examination in subjects with oligo or anovulation. RIA was based on hormone-specific antibodies that are immobilized to the wall of polypropylene tubes and the use of a ^125^I-labeled tracer. FAI was calculated as total T/SHBG ×100×3.467. The detection limit for total T, free T and SHBG were 0.10 ng/mL, 0.15 pg/mL and 0.04 nmol/L. Intra- and inter-assay coefficients of variation were 4.0–11.0% and 5.9–12.0% for total T, 4.0–17% and 8.0–18.3% for free T, and 2.8–5.3% and 7.9–8.5% for SHBG. All measures were performed at a single center (Seoul National University Hospital).

### Definition of metabolic syndrome

MetS was diagnosed if three or more of the following risk factors were present: waist circumference ≥80 cm; triglycerides ≥150 mg/dL; HDL cholesterol <50 mg/dL; fasting plasma glucose ≥100 mg/dL; systolic blood pressure ≥130 mmHg or diastolic blood pressure ≥85 mmHg [8]. Abdominal obesity was defined using the International Diabetes Federation (IDF) criteria, and triglycerides, HDL cholesterol, fasting plasma glucose and blood pressure were categorized according to the modified National Cholesterol Education Program (NCEP) Adult Treatment Panel (ATP) III guidelines [8].

### Statistical analysis

Distribution testing for normality was done using the Shapiro-Wilk test and data were log transformed to obtain normalized distributions. The baseline characteristics of the PCOS phenotype were expressed as means ± standard deviations (SD) or number (%) or age- and BMI-adjusted geometric means with 95% CIs. Statistical differences between PCO+HA+O and PCO+O (PCOS subjects with HA and without HA) groups were compared with independent sample *t*-tests or analysis of covariance (ANCOVA) for continuous variables and chi-square tests for categorical variables. Age- and BMI-adjusted geometric means for log-transformed variables are back-transformed for ease of interpretation and given with 95% CIs. The prevalence of MetS and metabolic syndrome parameters was compared by chi-squared tests. A multivariate logistic regression model was used to identify associations between the PCOS phenotype and MetS according to BMI. The odds ratios (ORs) of MetS according to age, BMI, and HA-associated parameters were also calculated using multivariable logistic regression analyses in non-obese and obese groups. Total T, free T, FAI, and mF-G scores were divided into low and high groups according to biochemical or clinical HA criteria. SHBG was categorized into two groups based on the median value. Values of *P*<0.05 were considered to indicate statistical significance. The data were analyzed using the SPSS software (version 19.0; SPSS Inc., Chicago, IL, USA).

## Results

### Baseline characteristics

The baseline characteristics and clinical, hormonal, and metabolic parameters of the PCOS subgroups are shown in Table 1. The mean age was 27.9±5.4 years and their mean BMI was 22.5±4.1 kg/m^2^. The mean age of the subjects in the PCO+O group was significantly higher than that of those in the PCO+HA+O group (*P* = 0.004); however, mean BMI was higher in the PCO+HA+O group (*P*<0.001). Of the 546 subjects, 26 (4.8%) had diabetes mellitus (DM) and PCO+HA+O group was more likely to have a history of DM (6.2% in the PCO+HA+O and 1.7% in the PCO+O, *P* = 0.023). After adjustment for age and BMI, higher levels of WC were observed in the PCO+O group (*P* = 0.003). Average systolic and diastolic BP values were similar in the PCOS subgroups, whereas FPG, PP2 glucose, fasting insulin, HOMA-IR, TC and LDL-C values were significantly higher in the PCO+HA+O group compared to those in the PCO+O group.

**Table 1:** Clinical, hormonal and metabolic parameters in the PCO+HA+O and PCO+O groups.

Variables (n = 700)	PCO+HA+O (n = 432)			PCO+O (n = 268)			*P* value
Age (years)^a^	27.4	±	5.5	28.6	±	5.1	0.004
BMI (kg/m^2^)^a^	23.1	±	4.3	21.5	±	3.4	<0.001
<25 kg/m^2b^	318	(73.6)		237	(88.4)		<0.001
≥25 kg/m^2b^	114	(26.4)		31	(11.6)		
WC (cm)^c^	75.9	(76.1–76.6)		77.4	(46.6–78.2)		0.003
Diabetes^bd^	23	(6.2)		3	(1.7)		0.023
Hypertension^b^	34	(7.9)		13	(4.9)		0.121
SBP (mmHg)^c^	112.4	(111.3–113.4)		113.1	(111.7–114.4)		0.385
DBP (mmHg)^c^	71.2	(70.2–72.1)		71.2	(70.0–72.4)		0.962
mF-G score^c^	5.1	(4.7–5.4)		2.4	(2.1–2.7)		<0.001
Total testosterone (ng/mL)^c^	0.70	(0.67–0.74)		0.35	(0.32–0.37)		<0.001
Free testosterone (pg/mL)^c^	0.91	(0.85–0.98)		0.62	(0.56–0.68)		<0.001
SHBG (nmol/L)^c^	58.4	(54.8–62.2)		62.7	(57.7–68.0)		0.188
Free androgen index^c^	4.2	(4.0–4.5)		1.9	(1.8–2.1)		<0.001
FPG (mg/dL)^c^	89.4	(88.0–90.7)		84.6	(82.9–86.3)		<0.001
PP2 glucose (mg/dL)^ce^	101.8	(98.7–105.0)		93.0	(89.5–96.8)		0.001
Fasting insulin (µU/mL)^c^	9.3	(8.8–9.8)		8.4	(7.8–9.0)		0.032
PP2 insulin (µU/mL)^cf^	34.2	(31.2–37.4)		32.5	(28.8–36.6)		0.512
HOMA-IR^c^	2.0	(1.9–2.2)		1.8	(1.6–1.9)		0.006
Hemoglobin A_1C_ (%)^cg^	5.4	(5.3–5.4)		5.5	(5.4–5.5)		0.108
TC (mg/dL)^c^	182.2	(179.3–184.9)		173.3	(170.0–176.8)		<0.001
HDL-C (mg/dL)^c^	56.5	(55.3–57.7)		57.0	(55.4–58.6)		0.646
LDL-C (mg/dL)^c^	101.9	(99.4–104.5)		93.3	(90.3–96.4)		<0.001
TG (mg/dL)^c^	92.3	(88.1–96.6)		86.4	(81.5–91.7)		0.093

Statistical differences between groups were compared with independent sample *t*-tests or ANCOVA for continuous variables, and chi-square tests for categorical variables.

a Data are shown as means ± SD;

b number (%).

c Values are age- and BMI-adjusted geometric means (95% CI) for log-transformed variables; adjusted means and 95% CIs were back-transformed. *P*-values were evaluated by ANCOVA, adjusting for age and BMI.

d Diabetes information was available for 546 participants (PCO+HA+O, n = 372; PCO+O, n = 174).

e PP2 glucose was available for 682 participants (PCO+HA+O, n = 422; PCO+O, n = 260).

f PP2 insulin was available for 646 participants (PCO+HA+O, n = 403; PCO+O, n = 243).

g Hemoglobin A_1C_ was available for 550 participants (PCO+HA+O, n = 372; PCO+O, n = 178).

BMI, body-mass index; WC, waist circumference; SBP, systolic blood pressure; DBP, diastolic blood pressure; mF-G, modified Ferriman–Gallwey; SHBG, sex hormone-binding globulin; FPG, fasting plasma glucose; PP2, postprandial 2 h; HOMA-IR, homeostatic model for insulin resistance; TC, total cholesterol; HDL-C, high density lipoprotein-cholesterol; LDL-C, low density lipoprotein-cholesterol; TG, triglyceride.

### Prevalence of metabolic syndrome parameters

MetS was more prevalent in the PCO+HA+O group (19.7%) than in the PCO+O (11.9%) group (*P* = 0.008). Increased WC was the most frequent abnormality in both PCOS subgroups (40.0% in the PCO+HA+O group, 34.7% in the PCO+O group, *P* = 0.157), followed by low HDL-C (32.6% in the PCO+HA+O group, 24.6% in the PCO+O group, *P* = 0.024). The prevalence of elevated FPG was much higher in PCOS patients with HA (*P* = 0.001). Although the mean SBP and DBP were not different, elevated BP was more frequent in patients with HA (*P* = 0.028) (Table 2).

**Table 2:** Prevalence of metabolic syndrome parameters according to PCOS subgroup.

Variables	PCO+HA+O (n = 432)		PCO+O (n = 268)		*P* value
WC ≥80 cm	173	(40.0)	93	(34.7)	0.157
TG ≥150 mg/dL	83	(19.2)	40	(14.9)	0.147
HDL-C <50 mg/dL	141	(32.6)	66	(24.6)	0.024
FPG ≥100 mg/dL	65	(15.0)	18	(6.7)	0.001
BP ≥130/85 mmHg	65	(15.0)	25	(9.3)	0.028
Metabolic syndrome (≥3 of the above factor)	85	(19.7)	32	(11.9)	0.008

Data are expressed as *n* (%) and differences evaluated by chi-square test.

### Results of multivariable logistic regression analysis of MetS

The results of multivariate logistic regression analysis of MetS in the two PCOS subgroups are presented in Table 3. We found statistically significant trends for an increased risk of MetS in the PCO+HA+O group compared to the PCO+O group after adjustment for age (*P* = 0.001); however, additional adjustment for BMI attenuated this association, rendering them statistically non-significant. For further analysis, PCOS subjects were divided into two groups: non-obese (BMI <25 kg/m^2^) and obese (BMI ≥25 kg/m^2^). When the study population was divided according to BMI, the MetS prevalence in the obese group were 58.1% and 46.5% in the PCO+O and PCO+HA+O groups, respectively. Among non-obese patients, the prevalence of MetS was much higher in subjects in the PCO+HA+O group compared to those in the PCO+O group (10.1% *vs*. 5.9%). After adjustment for age, the non-obese group with PCO+HA+O had an approximately two times higher risk of MetS compared with those with PCO+O (*P* = 0.021). However, it was no longer significant after adjustment for age and BMI. In contrast, the association between obese-PCOS and MetS was not significant in any of the adjusted models.

**Table 3:** Unadjusted and multivariate-adjusted odds ratios (OR) of metabolic syndrome according to PCOS subgroup.

Variables	Metabolic syndrome					
	Unadjusted OR (95% CI)		Age-adjusted OR (95% CI)		Age, BMI^a^-adjusted OR (95% CI)	
**All**						
PCO+O (n = 268)	1		1		1	
PCO+HA+O (n = 432)	1.807	(1.165–2.802)	2.121	(1.350–3.333)	1.198	(0.707–2.031)
*P*-value	0.008**		0.001**		0.502	
**BMI <25 kg/m^2^**						
PCO+O (n = 237)	1		1		1	
PCO+HA+O (n = 318)	1.782	(0.929–3.421)	2.192	(1.123–4.277)	1.683	(0.828–3.421)
*P*-value	0.082		0.021*		0.150	
**BMI ≥25 kg/m^2^**						
PCO+O (n = 31)	1		1		1	
PCO+HA+O (n = 114)	0.628	(0.281–1.400)	0.702	(0.308–1.601)	0.630	(0.269–1.480)
*P*-value	0.255		0.400		0.289	

*P<0.05, **P<0.01.

a Log-transformed value was used for analysis.

PCO, polycystic ovaries; HA, Hyperandrogenism; O, oligomenorrhea/amenorrhea.

### Odds ratio of MetS according to HA-associated parameters

Table 4 shows the ORs for MetS by age, BMI, and HA-associated parameters. According to multivariate logistic regression analysis, there were statistically significant associations between MetS and FAI and SHBG (data not shown) (*P* = 0.006 and *P*<0.001). Additionally, high FAI (>5.36) and low SHBG (≤70.0 nmol/L) (data not shown) were significantly associated with the risk of MetS in non-obese PCOS patients after adjustment for age, BMI, and HOMA-IR (*P*<0.001 and *P*<0.001). However, no associations were detected between MetS and FAI and SHBG (data snot shown) in obese PCOS subjects.

**Table 4:** Unadjusted and multivariate-adjusted odds ratios (OR) of metabolic syndrome according to age, BMI and HA-associated parameters.

Variables	Metabolic syndrome (All)							
	Unadjusted OR (95% CI)		Age-adjusted OR (95% CI)		Age, BMI^a^-adjusted OR (95% CI)		Age, BMI^a^, HOMA-IR^a^ adjusted OR (95% CI)	
**All**								
Age (years)	1.138	(1.087–1.191)***						
BMI (kg/m^2^)								
<25	1		1					
≥25	10.617	(6.811–16.549)***	10.645	(6.711–16.886)***				
Free androgen index								
≤5.36	1		1		1		1	
>5.36	4.256	(2.808–6.452)***	6.017	(3.808–9.508)***	2.616	(1.532–4.468)***	2.191	(1.255–3.826)**
mF-G score								
<6	1		1		1		1	
≥6	1.198	(0.787–1.826)	1.657	(1.061–2.587)*	1.108	(0.646–1.901)	1.086	(0.622–1.897)
**BMI <25 kg/m^2^**								
Age (years)	1.159	(1.079–1.246)***						
BMI (kg/m^2^)	1.967	(1.602–2.414)***	1.920	(1.559–2.365)***				
Free androgen index								
≤5.36	1		1		1		1	
>5.36	5.198	(2.775–9.739)***	8.381	(4.162–16.876)***	5.543	(2.623–11.716)***	4.234	(1.893–9.474)***
mF-G score								
<6	1		1		1		1	
≥6	1.150	(0.596–2.219)	1.747	(0.872–3.502)	1.899	(0.894–4.034)	1.886	(0.842–4.220)
**BMI ≥25 kg/m^2^**								
Age (years)	1.109	(1.039–1.183)**						
BMI (kg/m^2^)	1.126	(1.017–1.246)*	1.149	(1.032–1.279)*				
Free androgen index								
≤5.36	1		1		1		1	
>5.36	1.004	(0.521–1.933)	1.316	(0.653–2.651)	1.119	(0.542–2.312)	1.032	(0.492–2.167)
mF-G score								
<6	1		1		1		1	
≥6	0.610	(0.314–1.185)	0.784	(0.389–1.582)	0.721	(0.350–1.486)	0.716	(0.343–1.496)

*P<0.05, **P<0.01, ***P<0.001.

a Log-transformed value was used for analysis.

## Discussion

In this study, MetS was more prevalent in the PCO+HA+O group than in the PCO+O group. HA was significantly associated with MetS, especially in the non-obese PCOS subgroup (PCO+HA+O). Our data also indicated independent associations between HA-associated parameters and MetS in the non-obese subgroup. The clinical and demographical characteristics of subjects in the HA+PCO and HA+O groups are presented in Table S1.

The prevalence of obesity in women with PCOS has been reported to be 30–75% [1], [9]. Another study in Thailand showed that the prevalence of PCOS subjects with BMI ≥25 kg/m^2^ was approximately 50% [12]. In a multiracial group of women with PCOS, a mean BMI higher than 32 kg/m^2^ was reported, suggesting that obesity is strongly associated with PCOS [11], [19]. However, in our study, 20.7% were obese (BMI ≥25 kg/m^2^), and the prevalence in the non-obese group (BMI <25 kg/m^2^) was 88.4% in the PCO+O group and 73.6% in the PCO+HA+O group, suggesting that non-obese women with PCOS are common in our population. In addition, the mean BMI was about 22.5 kg/m^2^, which is similar to other Korean studies [14], [20]. Therefore, the reasons for the more common development of PCOS in non-obese Korean women should be elucidated in future studies.

In our study, the prevalence of MetS in the PCOS group varied across ethnic/racial regions and a high prevalence of MetS has been reported in Brazilian, Indian, Chinese and multiracial populations with PCOS [7]. The prevalence of MetS among females with POCS in the United States is 43.0–46.0% [11], [13], which is higher than age-matched females in the general population. In a previous study in young Korean females with PCOS, the prevalence of MetS was 14.5%, suggesting that MetS is less common in Korean patients with PCOS but higher than in the general population [14]. The BMI differences between different ethnic groups may have contributed to the above findings [7]. The overall prevalence of MetS in our study was 16.7%. Such a low prevalence may be related to the relatively low prevalence of obesity in our study population. The most frequent components among PCOS patients in the United States were high WC (67%) and low HDL-C (68%) [11]. Another study in Korea showed that the most frequent feature was low HDL-C (45%), followed by high WC (24%) [14]. Similarly in our subjects, the most prevalent factors of MetS was increased WC (38.0%), followed by low HDL-C (29.6%).

PCOS women have different metabolic risk according to phenotypes [21]. We found the prevalence of MetS to be significantly higher in the PCO+HA+O (19.7%) group than in the PCO+O (11.9%) group, as reported elsewhere [22]. Among Iranian women with PCOS, aged 18–42 years, the prevalence of MetS was higher in those with HA than in those without HA [22]. Moreover, after adjustment for age and BMI, FPG, PP2 glucose, fasting insulin, HOMA-IR, TC and LDL-C were significantly higher in the PCO+HA+O group than in the PCO+O group. Therefore, the significant differences between the groups regarding metabolic parameters may indicate an increased risk of metabolic and cardiovascular disease in the PCO+HA+O group. The prevalence of MetS components in the PCO+HA+O and PCO+O groups according to BMI are presented in Table S2.

The risk of MetS significantly increased among non-obese women with PCOS after adjustment for age. However, this association was not statistically significant after additional adjustment for BMI. As the sample size of our population was not large enough, additional adjustment might be attenuated the statistical power. In addition, androgen receptors have been found in both preadipocytes and adipocytes, and there is some evidence that sex steroid hormones play an important role in the adipose tissue metabolism [23]. Therefore, HA may be a key factor contributing to MetS, independent of or in synergy with central obesity and visceral adiposity [24]. Although the mechanisms linking these interactions are not clear, PCOS and associated metabolic comorbidities may possibly associated with a vicious circle represented by androgen excess and abdominal obesity [25]. The existing evidence suggests that androgen excess favors abdominal visceral adiposity, and visceral fat encourages further production of androgens through the direct effect of several mediators including hypoadiponectinemia, increased tumor necrosis factor-α (TNF-α), interleukin-6 (IL-6) and leptin levels and other factors, or as a result of insulin resistance and hyperinsulinims [25]. In the present study, the observed association between PCOS women with HA and MetS was attenuated after adjustment for age and BMI, suggesting that the relation could be partially mediated by BMI. There were statistically significant associations between MetS and SHBG and FAI in the PCO+HA+O subgroup among non-obese subjects. Because we evaluated biochemical HA according to levels of total T, free T, and FAI, it was thought that obese PCOS had no association with HA and MetS. That is, HA is associated with MetS in non-obese women but not obese women with PCOS.

Androgen excess in PCOS may involve the aggravation of metabolic abnormalities, such as increased visceral fat, decreased lipolysis in subcutaneous fat, insulin resistance in adipose tissue and skeletal muscle and lipid metabolism [16]. Additionally, androgens may contribute to potential direct vascular action [16]. However, the association with androgen-associated parameters and MetS in Korean women with PCOS has not been evaluated. In our study, we found a significant association with HA and MetS in the non-obese subjects, whereas this association was not observed in obese subjects. Moreover, non-obese PCOS showed the significant association with HA-associated parameters. Taking our findings into consideration, obesity itself may contribute to MetS among obese subjects with PCOS. Also, the proportion of obese subjects in this study population was relatively low. Thus, insignificant findings may be possibly explained by the lack of statistical power. Several studies provide epidemiologic evidence regarding HA and MetS in females with PCOS. Our results are similar to one such study, which reported that HA was a significant predictor of MetS in PCOS, independent of obesity and IR [19]. Moreover, studies of Italian adolescents with PCOS reported that HA was a risk factor for MetS, despite the subjects' relative youth [24]. In addition, HA was an independent risk factor for MetS among premenopausal women even without PCOS [26].

To our knowledge, this is the first study to evaluate the relationship between HA, its associated parameters, and MetS among Korean women with PCOS.

There are limitations to this study. First, as the study was cross-sectional in design, a causal relationship cannot be identified. Further longitudinal study will be needed to achieve higher levels of evidence. Second, although our results may be representative of PCOS patients in Korea, it may not be generalized to other ethnic groups. Third, total T, free T and SHBG were measured using a RIA. Although direct measurements by RIA assay might be useful clinically, this method is highly variable and inaccurate [27]. Alternatively, a gold standard method, such as liquid chromatography-tandem mass spectrometry, is particularly more accurate for clinical research [27]. Fourth, our results cannot reflect other potential confounding factors, such as smoking, drinking, exercise, and diet patterns. Accordingly, further studies with a follow-up will be needed to assess the present results.

## Conclusions

MetS was more prevalent in the PCO+HA+O group than in the PCO+O group, especially in the non-obese PCOS subjects. This study also demonstrate a significant association between MetS and HA, largely due to higher FAI and lower SHBG. Therefore, these findings imply greater risk of MetS in non-obese women with hyperandrogenemic PCOS phenotypes in Korea.

## Supporting Information

Table S1 **Mean values of clinical, hormonal and metabolic parameters in the HA+PCO and HA+O groups.**
(DOC)

Table S2 **Prevalence of metabolic syndrome factors in the PCO+HA+O and PCO+O groups.**
(DOC)
