# Supplementary material for: Prevalence of Metabolic Syndrome Is Higher among Non-Obese PCOS Women with Hyperandrogenism and Menstrual Irregularity in Korea
Source: PLoS One. 2014 Jun 5;9(6):e99252. doi: 10.1371/journal.pone.0099252 (PMC4047097; doi:10.1371/journal.pone.0099252)
Supplement: Table S1 — Mean values of clinical, hormonal and metabolic parameters in the HA+PCO and HA+O groups. (DOC) [file pone.0099252.s001.doc]

**Table S1.** Mean values of clinical, hormonal and metabolic parameters in the HA+PCO and HA+O groups.

| **Variables (n = 49)** | **HA+PCO (n = 20)** | | | **HA+O (n = 29)** | | |
| --- | --- | --- | --- | --- | --- | --- |
| Age (years) | 25.9 | ± | 5.9 | 28.2 | ± | 5.5 |
| BMI (kg/m^2^) | 21.6 | ± | 5.5 | 22.5 | ± | 4.1 |
| <25 kg/m^2^ | 17 | (85.0) | | 22 | (78.6) | |
| ≥25 kg/m^2^ | 3 | (15.0) | | 6 | (21.4) | |
| WC (cm) | 71.5 | ± | 13.2 | 74.8 | ± | 10.8 |
| Diabetes | 1 | (5.0) | | 1 | (3.6) | |
| Hypertension | 0 | (0.0) | | 0 | (0.0) | |
| SBP (mmHg) | 108.0 | ± | 11.2 | 108.2 | ± | 10.7 |
| DBP (mmHg) | 71.1 | ± | 8.4 | 68.8 | ± | 9.1 |
| mF-G score | 6.0 | (1-17) | | 6.0 | (0-20) | |
| Total testosterone (ng/mL) | 0.94 | ± | 0.48 | 0.80 | ± | 0.41 |
| Free testosterone (pg/mL) | 0.60 | ± | 0.60 | 0.90 | ± | 0.72 |
| SHBG (nmol/L) | 84.9 | ± | 41.7 | 90.8 | ± | 47.5 |
| Free androgen index | 5.2 | ± | 3.8 | 3.9 | ± | 2.6 |
| FPG (mg/dL) | 88.7 | ± | 9.4 | 87.4 | ± | 8.2 |
| PP2 glucose (mg/dL) | 99.3 | ± | 36.1 | 105.6 | ± | 27.3 |
| Fasting insulin (μU/mL) | 12.8 | ± | 17.5 | 9.5 | ± | 7.1 |
| PP2 insulin (μU/mL) | 39.9 | ± | 37.8 | 42.9 | ± | 39.1 |
| HOMA-IR | 2.7 | ± | 3.5 | 2.1 | ± | 1.8 |
| Hemoglobin A_1C_ (%) | 5.3 | ± | 0.39 | 5.3 | ± | 0.27 |
| TC (mg/dL) | 170.1 | ± | 27.6 | 178.6 | ± | 27.2 |
| HDL-C (mg/dL) | 60.7 | ± | 16.8 | 63.6 | ± | 18.5 |
| LDL-C (mg/dL) | 92.9 | ± | 21.5 | 99.0 | ± | 21.5 |
| TG (mg/dL) | 82.4 | ± | 31.7 | 80.2 | ± | 39.9 |

Data are expressed as means ± SD or number (%) or median (range).
