# Supplementary material for: Prevalence of Metabolic Syndrome Is Higher among Non-Obese PCOS Women with Hyperandrogenism and Menstrual Irregularity in Korea
Source: PLoS One. 2014 Jun 5;9(6):e99252. doi: 10.1371/journal.pone.0099252 (PMC4047097; doi:10.1371/journal.pone.0099252)
Supplement: Table S2 — Prevalence of metabolic syndrome factors in the PCO+HA+O and PCO+O groups. (DOC) [file pone.0099252.s002.doc]

**Table S2.** Prevalence of metabolic syndrome factors in the PCO+HA+O and PCO+O groups.

| **Metabolic syndrome factors (n)** | **All** | | | |
| --- | --- | --- | --- | --- |
|  | **PCO+HA+O (n=432)** | | **PCO+O (n=268)** | |
| 0 | 184 | (42.6) | 133 | (49.6) |
| 1 | 106 | (24.5) | 69 | (25.7) |
| 2 | 57 | (13.2) | 24 | (12.7) |
| 3 | 42 | (9.7) | 24 | (9.0) |
| 4 | 34 | (7.9) | 7 | (2.6) |
| 5 | 9 | (2.1) | 1 | (0.4) |
| **Metabolic syndrome (≥3 of the above factor)** | 85 | (19.7) | 32 | (11.9) |
| **Metabolic syndrome factors (n)** | **BMI <25 kg/m^2^** | | | |
|  | **PCO+HA+O (n=318)** | | **PCO+O (n=237)** | |
| 0 | 179 | (56.3) | 130 | (54.9) |
| 1 | 82 | (25.8) | 63 | (26.6) |
| 2 | 25 | (7.9) | 30 | (12.7) |
| 3 | 18 | (5.7) | 11 | (4.6) |
| 4 | 12 | (3.8) | 2 | (0.8) |
| 5 | 2 | (0.6) | 1 | (0.4) |
| **Metabolic syndrome (≥3 of the above factor)** | 32 | (10.1) | 14 | (5.9) |
| **Metabolic syndrome factors (n)** | **BMI ≥25 kg/m^2^** | | | |
|  | **PCO+HA+O (n=114)** | | **PCO+O (n=31)** | |
| 0 | 5 | (4.4) | 3 | (9.7) |
| 1 | 24 | (21.1) | 6 | (19.4) |
| 2 | 32 | (28.1) | 4 | (12.9) |
| 3 | 24 | (21.1) | 13 | (41.9) |
| 4 | 22 | (19.3) | 5 | (16.1) |
| 5 | 7 | (6.1) | 0 | (0.0) |
| **Metabolic syndrome (≥3 of the above factor)** | 53 | (46.5) | 18 | (58.1) |

Data are expressed as *n* (%).
